# The Japanese breast cancer society clinical practice guidelines for radiation treatment of breast cancer, 2022 edition

**DOI:** 10.1007/s12282-024-01568-4

**Published:** 2024-04-05

**Authors:** Michio Yoshimura, Chikako Yamauchi, Naoko Sanuki, Yasushi Hamamoto, Kimiko Hirata, Jiro Kawamori, Mariko Kawamura, Mami Ogita, Yutaka Yamamoto, Hiroji Iwata, Shigehira Saji

**Affiliations:** 1 Department of Radiation Oncology and Image-Applied Therapy, Kyoto University Graduate School of Medicine, 54 Shogoin-kawahara‑cho, Sakyo‑ku, Kyoto, 606‑8507 Japan; 2 Department of Radiation Oncology, Shiga General Hospital, Moriyama, Japan; 3 Department of Radiology, Yokkaichi Municipal Hospital, Yokkaichi, Japan; 4 Department of Radiation Oncology, National Hospital Organization Shikoku Cancer Center, Matsuyama, Japan; 5 Department of Radiation Oncology, Kyoto City Hospital, Kyoto, Japan; 6 Department of Radiation Oncology, St. Luke’s International Hospital, Tokyo, Japan; 7 Department of Radiology, Nagoya University Graduate School of Medicine, Nagoya, Japan; 8 Department of Radiology, The University of Tokyo Hospital, Tokyo, Japan; 9 Department of Breast and Endocrine Surgery, Kumamoto University Graduate School of Medical Science, Kumamoto, Japan; 10 Department of Breast Oncology, Aichi Cancer Center Hospital, Nagoya, Japan; 11 Department of Medical Oncology, Fukushima Medical University, Fukushima, Japan

**Keywords:** Clinical practice guideline, Clinical question, Radiation therapy, Radiotherapy, Breast cancer

## Abstract

The Breast Cancer Clinical Practice Guidelines, organized by the Japanese Breast Cancer Society (JBCS), were published in 2022. We present the English version of the Radiation Therapy (RT) section of the guidelines. The JBCS formed a task force to update the 2018 version of the JBCS Clinical Practice Guidelines. The Background Questions (BQs) contain the standard treatments for breast cancer in clinical practice, whereas the Clinical Questions (CQs) address daily clinical questions that remain controversial. Future Research Questions (FRQs) explore the subjects that are considered important issues, despite there being insufficient data for inclusion as CQs. The task force selected the 12 BQs, 8 CQs, and 6 FRQs for the RT section. For each CQ, systematic literature reviews and meta-analyses were conducted according to the Minds Manual for Guideline Development 2020, version 3.0. The recommendations, strength of recommendation, and strength of evidence for each CQ were determined based on systematic reviews and meta-analyses, and finalized by voting at the recommendation decision meeting.

## Introduction

The Japanese Breast Cancer Society (JBCS) Clinical Practice Guideline was revised in 2022. In the 2022 edition,

the concepts and the methods used for the guideline were based on the Minds Manual for Guideline Development 2020, version 3.0. In this article, we list all background questions (BQs, Table 1) and Clinical Questions (CQs; Table 2) of Radiation Therapy (RT), describe the revised points, and provide a short explanation.

**Table 1:** Background Questions

Postoperative Radiotherapy for Breast Cancer	
BQ1	Is whole breast irradiation (WBI) following breast-conserving surgery (BCS) recommended for patients with stage I–II breast cancer?
Statement	WBI is the standard treatment
BQ2	Is RT recommended for patients with DCIS after BCS?
Statement	WBI is the standard treatment
BQ3	Is RT following BCS recommended for patients with a pathologic complete response (pCR) after neoadjuvant chemotherapy?
Statement	WBI is the standard treatment
BQ4	Is regional nodal irradiation (RNI) to the supraclavicular region recommended for patients with ≥ 4 positive axillary nodes after BCS?
Statement	RT to the ipsilateral supraclavicular node is the standard treatment
BQ5	Is PMRT recommended for patients with ≥ 4 positive axillary nodes after mastectomy?
Statement	PMRT is the standard treatment
BQ6	Is irradiation to the chest wall and supraclavicular lymph nodes recommended for PMRT?
Statement	Irradiation to the chest wall and supraclavicular lymph nodes is the standard treatment
BQ7	Is PMRT recommended for node-negative patients with large tumors or for those with involved resection margins after mastectomy?
Statement	PMRT should be considered for node-negative patients with large tumors (T3‐4). Irradiation to the chest wall is acceptable only if the patient has appropriate axillary lymph node evaluation and no risk factors other than a large tumor PMRT should be considered for node-negative patients with involved resection margins. Irradiation to the chest wall is acceptable only if the patient has no risk factors other than involved margins
BQ8	
BQ8a	Is PMRT recommended for the patients with autologous breast reconstruction?
Statement	PMRT is the standard treatment for patients requiring it, but PMRT may increase adverse events in patients with autologously reconstructed breasts
BQ8b	Is PMRT recommended for the patients with prosthetic breast reconstruction?
Statement	Although PMRT is the standard treatment for patients requiring it, the risk of adverse events and impairment of cosmesis due to PMRT should be thoroughly discussed with patients before surgery
BQ8c	What is the appropriate timing for PMRT in patients undergoing two-stage prosthetic breast reconstruction?
Statement	Although it is preferable to perform PMRT after exchanging a tissue expander (TE) to an implant, if necessary, PMRT is acceptable for a patients with a TE
BQ9	What is the appropriate timing for RT after breast surgery?
Statement	・RT should be initiated within 20 weeks of surgery in patients who do not receive postoperative chemotherapy ・Chemotherapy should be completed before RT in patients requiring postoperative chemotherapy ・Concurrent chemoradiotherapy should be avoided in patients in adjuvant settings ・Endocrine therapy may be administered concurrently with RT ・Although anti-HER2 therapy may be administered concurrently with RT, patients with left-sided breast cancer should be carefully monitored to prevent adverse cardiac events
BQ10	Is RT recommended after breast surgery for patients with breast cancer with *BRCA* pathogenic variants?
Statement	RT after BCS is the standard treatment PMRT according to the clinical indications is the standard treatment
Radiotherapy for Metastatic/Recurrent Breast Cancer	
BQ11	Is RT recommended for painful bone metastases of breast cancer?
Statement	RT is the standard treatment for painful bone metastases
BQ12	Is RT recommended for brain metastasis of breast cancer?
Statement	RT is the standard treatment

**Table 2:** Clinical Questions

		SoR	SoE	Consensus rate
Postoperative Radiotherapy for Breast Cancer				
CQ1	Is hypofractionated WBI recommended as an equivalent treatment to conventionally fractionated WBI?			
Recommendation	For patients aged ≥ 50 years, with pT1-2N0 without chemotherapy, hypofractionated WBI is strongly recommended	1	Strong	96%
	For patients other than those with the above three criteria, hypofractionated WBI is strongly recommended	1	Moderate	85%
	For patients with DCIS, hypofractionated WBI is strongly recommended	1	Weak	77%
CQ2	Is boost irradiation to the tumor bed following WBI recommended for patients with negative surgical margins after BCS?			
Recommendation	Tumor bed boost irradiation is weakly recommended for patients with pathologically negative margins after BCS for invasive breast cancer	2	Moderate	94%
CQ3	Is accelerated partial breast irradiation (APBI) recommended after BCS?			
Recommendation	APBI is weakly recommended under the following conditions ・For older low-risk patients, either as a clinical trial or at facilities that are skilled in irradiation techniques, with adequate quality control ・Intraoperative RT should be performed in patients who request it after being informed that the local recurrence rate is higher than that of WBI and there is no difference in the OS rate	2	Moderate	92%
CQ4	Is regional nodal irradiation (RNI) to the supraclavicular region recommended for patients with 1–3 positive axillary nodes after BCS with axillary dissection?			
Recommendation	RNI (supraclavicular region) is weakly recommended	2	Weak	98%
CQ5	Is PMRT recommended for patients with 1‐3 positive axillary nodes after mastectomy with axiallay dissection			
Recommendation	PMRT is weakly recommended	2	Moderate	71%
CQ6	Is it recommended to include the internal mammary nodes (IMNs) in patients with positive axillary nodes after breast surgery who undergo RNI or PMRT?			
Recommendation	It is weakly recommended to include IMNs	2	Weak	100%
Radiotherapy for Metastatic/Recurrent Breast Cancer				
CQ7	Is single-fraction RT of 8 Gy recommended as an equivalent treatment to multifraction RT for pain relief in patients with painful bone metastases of breast cancer?			
Recommendation	Single-fraction RT of 8 Gy is strongly recommended for pain relief in patients with painful bone metastases of breast cancer	1	Moderate	90%
CQ8	Is the addition of whole-brain irradiation recommended after stereotactic radiosurgery (SRS) for 1–4 brain metastases of breast cancer measuring < 3 cm			
Recommendation	It is weakly recommended not to add whole-brain irradiation after SRS	3	Moderate	98%

### Postoperative radiation therapy for breast cancer

**BQ1**. Is whole breast irradiation (WBI) following breast-conserving surgery (BCS) recommended for patients with stage I–II breast cancer?

#### Statement

WBI is the standard treatment.

**BQ2**. Is RT recommended for patients with ductal carcinoma in situ (DCIS) after BCS?

#### Statement

WBI is the standard treatment.

**BQ3.** Is RT following BCS recommended for patients with a pathologic complete response (pCR) after neoadjuvant chemotherapy?

#### Statement

WBI is the standard treatment.

**CQ1.** Is hypofractionated WBI recommended as an equivalent treatment to conventionally fractionated WBI?

#### Recommendation

・For patients aged ≥ 50 years, with pT1-2N0 without chemotherapy, hypofractionated WBI is strongly recommended [Strength of recommendation (SoR): 1; strength of evidence (SoE): strong; consensus rate; 96% (45/47)].

・For patients other than those with the above three criteria, hypofractionated WBI is strongly recommended [SoR: 1, SoE: moderate, consensus rate: 85% (41/48)].

・For patients with DCIS, hypofractionated WBI is strongly recommended [SoR: 1; SoE: weak; consensus rate: 77% (36/47)].

Regarding the dose and fractionation for WBI, a total dose of 45–50.4 Gy in 25–28 fractions over a period of 4.5–5.5 weeks has conventionally been used. Based on the results of randomized controlled trials (RCTs) performed in Canada [1] and the United Kingdom [2], hypofractionated WBI for approximately 3 weeks was used for many patients with conserved breasts instead of conventional fractionation. In 2011, the American Society for Radiation Oncology (ASTRO) guidelines stated that for patients aged ≥ 50 years, with pT1-N0 after BCS, no systemic chemotherapy, and dose homogeneity within ± 7% in the central axis plane, hypofractionated WBI is equivalent to conventionally fractionated WBI, and hypofractionated irradiation is not contraindicated in other patients [3]. Subsequently, in the updated 2018 guidelines, which included new evidence, restrictions such as age limits and the use of systemic chemotherapy were removed and hypofractionated irradiation was recommended for all patients not requiring regional lymph node irradiation (RNI) [4]. However, dose homogeneity was required to minimize the area by more than 105% in three dimensions; other factors, such as the use of the field in field method and image-guided position matching in patients with large setup errors, were also required. In the present revision, we conducted a systematic review of RCTs on hypofractionation. Compared with conventional fractionation, the risk ratios (RRs) for hypofractionation were not significantly different for local recurrence (RR 0.94, 95% CI 0.79–1.11) and overall survival (OS) (HR 0.92, 95% CI 0.82–1.04). Results from systematic reviews suggest that the treatment effects are comparable and adverse events tend to be similar or milder with hypofractionation. A follow-up period > 10 years is necessary to assess late adverse events, particularly ischemic heart disease. However, it has been reported that the incidence of ischemic heart disease with hypofractionated irradiation is low and does not increase over a 10-year period compared with that of conventionally fractionated irradiation [2]. Therefore, hypofractionated irradiation is recommended, considering dose homogeneity and the dose to normal tissues, such as the heart. However, it is possible that the number and severity of adverse events caused by hypofractionated irradiation may differ in Japanese patients due to racial differences and body size. Therefore, the JCOG (Japan Clinical Oncology Group) 0906 trial was conducted involving a single arm of over 300 patients with invasive breast cancer who had undergone breast-conserving surgery, presenting with a clinical tumor size of less than 3 cm, a clear resection margin, and pN0-1c. The study reported that hypofractionated irradiation can be safely performed in Japanese patients with acceptable acute and late effects in normal tissues at a median follow-up of 70.5 months [5]. The ASTRO guidelines [4] and the NCCN guidelines [6] state that hypofractionated irradiation is the standard treatment in all cases. The JBCS guideline committee decided to expand the recommendations and indications from the 2020 edition of the guidelines and recommended hypofractionated irradiation as an equivalent treatment to conventionally fractionated irradiation for WBI in all cases. We also examined the indications for hypofractionated irradiation for DCIS. Observational studies have shown that local control rates are comparable to those of conventionally fractionated irradiation [7]. The DBCG HYPO study from Denmark, which included DCIS in 13% (123 patients) reported no difference between the hypofractionated and conventionally fractionated groups regarding the local recurrence rate [8]. For DCIS, we recommend the use of hypofractionated irradiation as an equivalent treatment to conventional fractionated irradiation, as shown in the 2018 ASTRO [4] and NCCN [6] guidelines. In summary, the effects and adverse events of hypofractionated and conventionally fractionated WBI appear to be equivalent. In addition, hypofractionated WBI is not time-consuming and is cost effective. Therefore, after considering dose homogeneity and dose to normal tissues, hypofractionated WBI is recommended in all cases, including DCIS.

**CQ2.** Is boost irradiation to the tumor bed following WBI recommended for patients with negative surgical margins after BCS?

#### Recommendation

Tumor bed boost irradiation is weakly recommended for patients with pathologically negative margins after BCS for invasive breast cancer [SoR: 2; SoE: moderate; consensus rate: 94% (45/48)].

In patients who underwent pathologically complete excision for invasive disease, an RCT conducted by the European Organization for Research and Treatment of Cancer (EORTC) revealed that delivering a 16-Gy boost to the tumor bed reduced the rate of ipsilateral breast tumor recurrence (IBTR) from 16.4 to 12.0% but did not improve OS [9].

A meta-analysis of IBTR and OS was performed, and boost irradiation significantly reduced the incidence of IBTR (RR 0.66, 95% CI 0.57–0.77, *p* < 0.0001), and OS was not significantly different between patients with and without boost irradiation (HR 0.96, 95% CI 0.71–1.31, *p* = 0.81). In the EORTC trial, there was a significant decrease in the local recurrence rate in the boost group for patients aged ≤ 40 years, 41–50 years, 51–60 years, and ≥ 61 years; the absolute risk reduction was particularly significant in younger patients [9, 10]. However, no improvement in OS was observed; therefore, the risks must be assessed to determine the indications. A meta-analysis of long-term cosmesis was performed, and boost irradiation significantly worsened cosmesis (RR 1.99, 95% CI 1.59–2.49, *p* < 0.0001). The EORTC reported a significantly higher frequency of breast fibrosis in the boost group. However, the frequency of severe fibrosis was lower (5.2%) at 20 years, and there was no significant difference in the frequency of severe fibrosis between the boost and non-boost groups in patients < 40 years old [9]. Tumor bed boost irradiation is weakly recommended for invasive breast cancer with negative pathological margins after BCS, particularly in younger patients, considering the prolonged treatment time and costs. In addition, several non-RCTs have investigated the usefulness of boost irradiation for local control in DCIS [11] [12]; however, no consensus has been reached regarding the usefulness of boost irradiation for DCIS.

**CQ3**. Is accelerated partial breast irradiation (APBI) recommended after BCS?

#### Statement

APBI is weakly recommended under the following conditions [SoR: 2; SoE: moderate; consensus rate: 92% (35/38)].

・APBI should be performed for older low-risk patients, either as a clinical trial or at facilities that are skilled in irradiation techniques, with adequate quality control.

・Intraoperative RT should be performed in patients who request it after being informed that the local recurrence rate is higher than that of WBI and there is no difference in the OS rate.

Recently, the long-term follow-up results of several RCTs on APBI have been published [13–16]. According to the Cochrane systematic review of partial breast irradiation in 2021 [17], local recurrence-free survival was slightly worse in the PBI group than in the WBI group, but the local recurrence rate was low and the difference was small, with 30% of the cases reported for intraoperative RT, which tended to cause more local recurrences; caution is required in interpreting the results. No significant differences were observed in the OS, cause-specific survival, or distant metastasis-free survival rates. We conducted a meta-analysis of RCTs including all APBI treatment modalities (intraoperative RT, external-beam RT, and brachytherapy), which showed that the local recurrence rate was significantly higher in the APBI group than in the WBI group (RR 1.81, 95% CI 1.16–2.84, p = 0.009). In a sub-analysis, a meta-analysis for intraoperative irradiation showed that the local recurrence rate was significantly higher in the APBI group than in the WBI group (RR 3.38, 95% CI 2.14–5.35, *p* < 0.00001), but a meta-analysis for external beam or brachytherapy showed that there was no significant difference in the local recurrence rate between APBI and WBI groups (RR 1.23, 95% CI 0.97–1.57, *p* = 0.09). Meta-analyses for OS and for distant recurrence rate showed no significant differences between the APBI and WBI groups for OS (HR 1.00, 95% CI 0.87–1.14, *p* = 0.98); and distant recurrence rate: (HR 0.94, 95% CI 0.74–1.20, *p* = 0.63). Meta-analyses showed no significant differences in cosmesis and late skin toxicity between the APBI and WBI groups (cosmesis: RR 1.21, 95% CI 0.73–1.99; late skin toxicity: *p* = 0.46, RR 1.58, 95% CI 0.33–7.52, *p* = 0.56), but showed that the incidence of fat necrosis was significantly higher in the APBI group (RR 2.80, 95% CI 1.16–6.78, *p* = 0.02). Therefore, APBI should be performed as a clinical trial or at facilities skilled in irradiation techniques, with adequate quality control, after appropriate patient selection and verification of treatment accuracy. Furthermore, intraoperative irradiation should be administered to patients who desire it after they have been informed that the local recurrence rate is higher than that of WBI and that there is no difference in OS.

**BQ4**. Is RNI to the supraclavicular region recommended for patients with four or more positive axillary nodes after BCS?

#### Statement

RT to the ipsilateral supraclavicular node is the standard treatment.

**CQ4**. Is RNI to the supraclavicular region recommended for patients with 1–3 positive axillary nodes after BCS with axillary dissection?

#### Recommendation

RNI (supraclavicular region) is weakly recommended [SoR: 2; SoE: weak; consensus rate: 98% (47/48)].

Of the RCTs examining the benefit of postoperative RT including RNI, two studies included patients treated with BCS: the MA.20 trial, which included 1,832 patients, of whom 85% had 1–3 positive LNs [18], and the EORTC (22,922/10925) trial, in which 76% of 4004 patients underwent BCS and 43% had 1–3 metastatic nodes [19, 20]. The results revealed no significant difference in OS in either trial or inconsistent results regarding disease-free survival, distant metastasis-free survival, and breast cancer mortality between the two trials. A combined meta-analysis of the two studies revealed that postoperative RT including RNI significantly reduced breast cancer mortality (HR 0.81, 95% CI 0.71–0.92, *p* = 0.001), but did not reduce regional nodal recurrence or distant metastases, or improve disease-free survival or OS. For the adverse events evaluated in the two studies, lymphedema was significantly higher in the postoperative irradiation group including RNI from 4.5% to 8.4% in the MA.20 study (*p* = 0.001) and from 10.5 to 12.0% in the EORTC (22,922/10925) study. When combined, there was a trend toward increased lymphedema but with no statistically significant difference (RR 1.42, 95% CI 0.89–2.26, *p* = 0.14) for RNI. Regarding secondary malignancy, a combined analysis of the two studies showed no significant increase (RR 0.98, 95% CI 0.86–1.12, *p* = 0.82). However, long-term observational data are required to confirm these adverse events. Unlike after total mastectomy, almost all patients with BCS should undergo postoperative WBI. The increase in treatment costs and hospital visits are minimal when RNI is added. Moreover, the evaluated studies were initiated before the widespread use of modern systemic agents, such as anti-HER2 therapy, taxanes, and aromatase inhibitors, with an increased contribution of each to the outcomes, including locoregional control, the relative significance of RT may possibly be decreasing. The indication for RNI is not determined solely by the number of LN metastases and should be based on an overall consideration of other risks (e.g., large tumor size, high histological grade, hormone insensitivity, positive lymphovascular invasion, and medial or central location of the primary tumor). It is advisable that the patient and physician discuss and decide on a policy based on a risk/benefit analysis. From the above findings, adding RNI (supraclavicular region) to WBI is weakly recommended.

**BQ5**. Is postmastectomy radiation therapy (PMRT) recommended for patients with four or more positive axillary nodes after mastectomy?

#### Statement

PMRT is the standard treatment.

**CQ5**. Is PMRT recommended for patients with 1–3 positive axillary nodes after mastectomy with axillary dissection?

#### Recommendation

PMRT is weakly recommended [SoR: 2; SoE: moderate; consensus rate: 71% (34/48)].

Of the 22 RCTs involving PMRT, the EBCTCG (Early Breast Cancer Trialists’ Collaborative Group) meta-analysis included 1314 patients with 1–3 positive axillary LNs [21]. PMRT reduced the 10-year locoregional recurrence rate from 20.3 to 3.8% (rate ratio 0.24, 95% CI 0.17−0.34, 2*p* < 0.00001), the 10-year overall recurrence rate from 45.7 to 34.2% (rate ratio 0.68, 95% CI 0.57−0.82, 2p = 0.0006), and the 20-year breast cancer mortality rate from 50.2 to 42.3% (rate ratio 0.80, 95% CI 0.67−0.95, 2p = 0.01). Although the 20-year all-cause mortality was 53.5% in patients who received PMRT, compared with 56.5% in those who did not receive PMRT, the difference was not statistically significant (rate ratio 0.89, 95% CI 0.77−1.04, 2p > 0.1). The RCTs included in the meta-analysis were conducted before modern systemic treatments, such as aromatase inhibitors, anti-HER2 therapy, and taxane-based chemotherapy became widespread. Thus, it should be noted that the relative significance of PMRT may be declining. We conducted a meta-analysis of observational studies in patients with 1−3 positive axillary LNs who were treated with anthracycline-and/or taxane-based chemotherapy. The meta-analysis revealed that PMRT significantly decreased the locoregional recurrence rate (HR 0.37, 95% CI 0.27−0.51, *p* < 0.00001); however, there was no difference in distant recurrence (HR 0.89, 95% CI 0.71−1.11, *p* = 0.30), and in breast cancer mortality (HR 0.98, 95% CI 0.90−1.06, *p* = 0.60), with or without PMRT; the OS rate was higher in the PMRT group (HR 0.83, 95% CI 0.70−0.98, *p* = 0.03). Late adverse heart disease was also assessed in a systematic review [22]. Although a long-term follow-up was performed, it is necessary to be cognizant that old irradiation techniques were used in the studies included in the systematic review. Based on this review, the mortality rate of heart disease without breast cancer recurrence was higher in the radiation group, 0.36% vs. 0.30% in the non-radiation group (rate ratio 1.30, 95% CI 1.15−1.46, *p* < 0.001). Secondary malignancy events were assessed in two systematic reviews. It should be noted that the reviews included patients who underwent lumpectomy and that the irradiation fields were not uniform, the incidence of all secondary cancers except breast cancer increased in the RT group in both reviews (rate ratio 1.23, 95% CI 1.12−1.36, *p* < 0.001; relative risk 1.22, 95% CI 1.06−1.41, *p* = 0.005) [22] [23]. Taylor et al. reported that the incidence of secondary cancers, excluding breast cancer, was 0.50% in the irradiated group and 0.42% in the non-irradiated group. For arm lymphedema, only one prospective cohort study was identified as being highly directed. In the study, although PMRT increased the cumulative risk of lymphedema from 18.3 to 23.9% at two years after surgery, the difference was not statistically significant (rate ratio 1.29, 95% CI 0.71−2.35, *p* = 0.40) [24]. However, meta-analyses have shown that irradiation increases lymphedema compared to non-irradiation and that RNI increases lymphedema; hence, the possibility of an increase in lymphedema with PMRT should be noted. Skin and lung toxicities were reported in the EORTC 22922/10925 trial [25, 26]. For patients who underwent RNI, skin toxicities occurred in 13.6%, radiation pneumonitis occurred in 0.7% in three years and pulmonary fibrosis was observed in 5.7% in 15 years. Various guidelines strongly recommend PMRT [6, 27], but there may be subgroups of patients who should not undergo PMRT. The ASCO/ASTRO/SSO guidelines state that several factors may decrease the risk of locoregional recurrence or increase the risk of PMRT-related complications. Although there is no consensus regarding which subgroups of patients cannot undergo PMRT, patients may choose not to undergo PMRT after comprehensive risk evaluation.

**BQ6**. Is irradiation to the chest wall and supraclavicular LNs recommended for PMRT?

#### Statement

Irradiation to the chest wall and supraclavicular LNs is the standard treatment.

**BQ7**. Is PMRT recommended for node-negative patients with large tumors or for those with involved resection margins after mastectomy?

#### Statement

PMRT should be considered for node-negative patients with large tumors (T3−4). Irradiation to the chest wall only is acceptable if the patient has appropriate axillary LN evaluation and no risk factors other than a large tumor.

PMRT should be considered for node-negative patients with involved resection margins. Irradiation to the chest wall is acceptable only if the patient has no risk factors other than the involved margins.

**BQ8**. Is PMRT recommended for the patients who underwent mastectomy and breast reconstruction?

**BQ8a**. Is PMRT recommended for the patients with autologous breast reconstruction?

#### Statement

PMRT is the standard treatment for patients requiring it, but PMRT may increase adverse events in patients with autologously reconstructed breasts.

**BQ8b**. Is PMRT recommended for the patients with prosthetic breast reconstruction?

#### Statement

Although PMRT is the standard treatment for patients requiring it, the risk of adverse events and impairment of cosmesis due to PMRT should be thoroughly discussed with patients before surgery.

**BQ8c**. What is the appropriate timing for PMRT in patients undergoing two-stage prosthetic breast reconstruction?

#### Statement

Although it is preferable to perform PMRT after exchanging a tissue expander (TE) to an implant, if necessary, PMRT is acceptable for a patient with a TE.

**CQ6**. Is it recommended to include the internal mammary nodes (IMNs) in patients with positive axillary nodes after breast surgery who undergo RNI or PMRT?

#### Recommendation

It is weakly recommended to include IMNs [SoR: 2; SoE: weak; consensus rate: 100% (48/48)].

Although there is consensus regarding the inclusion of supraclavicular LNs when performing RNI, there is no consensus on whether IMNs should be included. Although the frequency of IMN recurrence is low even when RNI is not performed, IMNs have been included in the irradiated field in the major RCTs [28–31] that showed improved survival with PMRT. Two RCTs examined the benefits of IMN irradiation (IMNI): the French trial [32] and the KROG 08–06 trial [33]. The French trial included 1334 patients with positive axillary LN metastases or primary lesions in the inner/medial area. The median follow-up period was 11.3 years and there was no significant difference in the 10-year survival rates. However, it should be considered that approximately 85% of the patients had T1–2 tumors, and approximately 25% had no LN metastasis. The KROG 08–06 trial, which included 747 patients with positive LN metastasis, showed no statistical differences in the 7-year disease-free survival, breast cancer mortality, and OS rates between IMNI and non-IMNI groups. However, a significant benefit was observed with IMNI in 7-year disease-free survival (91.8% vs. 81.6%, HR 0.42, 95% CI 0.22–0.82, *p* = 0.008), and breast cancer mortality (4.9% vs. 10.2%, HR 0.41, 95% CI 0.17–0.99, *p* = 0.04) in patients with medial/central tumors. RCTs comparing irradiation of the breast or chest wall with or without RNI (EORTC 22922/10925 trial [20] and MA. 20 trial [18]), included the IMNs in the irradiated field in the RNI group. The DBCG-IMN trial, which included 3089 patients with positive LNs, reported the results of IMNI in addition to breast or chest wall and supraclavicular node irradiation in patients with right-sided cancer, and the results of no IMNI in patients with left-sided breast cancer [34]. Approximately 35% of the patients underwent BCS, and the remaining 65% underwent mastectomy. At a median follow-up of 8.9 years, the OS rate improved significantly from 72.2% in the non-irradiated group to 75.9% in the irradiated group (HR 0.82, *p* = 0.005). With IMNI, the breast cancer mortality rate also decreased significantly (20.9% vs. 23.4%, HR 0.85, *p* = 0.03), and distant metastasis rates also tended to decrease (27.4% vs. 29.7%, HR 0.89, *p* = 0.07) but the difference was not statistically significant. In particular, IMNI improved the OS rate in patients with four or more axillary LN metastases. We conducted a meta-analysis of observation studies and this prospective cohort study, which showed that there was no statistically significant difference in locoregional recurrence (RR 0.74, 95% CI 0.48–1.14, *p* = 0.17) and distant recurrence (RR 0.91, 95% CI 0.81–1.02, *p* = 0.09) with or without IMNI; breast cancer mortality was lower in the IMNI group (HR 0.87, 95% CI 0.77–0.98, *p* = 0.02), and OS improved with IMNI (HR 0.83, 95% CI 0.76–0.91, *p* < 0.0001). A meta-analysis of the French trial [32], EORTC 22922/19025 trial [20], and MA.20 trial [18] showed a trend toward an increase in heart disease in the IMNI group; however, the difference was not statistically significant. In the KROG 08–06 trial, IMNI resulted in a higher rate of grade 1/2 radiation pneumonitis, but the difference was not statistically significant (6.1% in IMNI group vs. 3.2% in the non-IMNI group, *p* = 0.06) [33]. In the EORTC 22922/19025 trial, any grade of lung fibrosis occurred in 5.7% of RNI group [26]. Although IMNI is not recommended for all patients undergoing RNI, it should be performed in high-risk patients. Although the evidence is insufficient, clinically or pathologically positive IMN metastases, 4 or more positive axillary LNs, or axillary LN metastases from a medial or central primary tumor are considered high-risk groups [33–35].

**BQ9.** What is the appropriate timing for RT after breast surgery?

#### Statement

・RT should be initiated within 20 weeks of surgery in patients who do not receive postoperative chemotherapy.

・Chemotherapy should be completed before RT in patients requiring postoperative chemotherapy.

・Concurrent chemoradiotherapy should be avoided in patients in adjuvant settings.

・Endocrine therapy may be administered concurrently with RT.

・Although anti-HER2 therapy may be administered concurrently with RT, patients with left-sided breast cancer should be carefully monitored to prevent adverse cardiac events.

(In recent years, several new agents have been incorporated into perioperative treatment, and the feasibility of combining them with RT should be noted by referring to the latest information).

**BQ10**. Is RT recommended after breast surgery for patients with breast cancer with *BRCA* pathogenic variants?

#### Statement

RT after BCS is the standard treatment.

PMRT according to the clinical indications is the standard treatment.

### Radiation therapy for metastatic or recurrent breast cancer

**BQ11**. Is RT recommended for painful bone metastasis of breast cancer?

#### Statement

RT is the standard treatment for painful bone metastases.

**CQ7**. Is single-fraction RT of 8 Gy recommended as an equivalent treatment to multifraction RT for pain relief in patients with painful bone metastases of breast cancer?

#### Recommendation

Single-fraction RT of 8 Gy is strongly recommended for pain relief in patients with painful bone metastases of breast cancer [SoR: 1; SoE: moderate; consensus rate: 90% (43/48)].

Palliative RT with multifraction RT (e.g., 30 Gy in 10 fractions) has long been used for painful bone metastases; however, systematic reviews [36–39] have demonstrated the efficacy and safety of single-fraction RT of 8 Gy for painful bone metastases, and several recent guidelines (ASTRO, WHO) state that single-fraction RT is useful for painful bone metastases [40, 41]. We conducted a meta-analysis of relatively large RCTs (> 100 cases in both single-and multifraction groups) to evaluate the usefulness of single-fraction RT of 8 Gy for painful bone metastases.

Pain relief rates in the single-fraction and multifraction groups were similar (56.8% vs. 57.2%, RR 1.00, 95% CI 0.95–1.05, *p* = 0.97). The rates of spinal cord compression and pathological fracture were also similar. However, the re-irradiation rate of the single-fraction group was higher than that of the multifraction group (20% vs. 8%, RR 2.36, 95% CI 1.65–3.38, *p* < 0.00001). There was a trend toward lower incidence of acute adverse events ≥ grade 2 in the single-fraction group (13.9% vs. 20.0%, RR 0.73, 95% CI 0.54–1.00, *p* = 0.05). Single-fraction RT of 8 Gy, which is more convenient and economical for patients, provides pain relief equivalent to multifraction RT without the additional harm of acute adverse events. Therefore, single-fraction RT of 8 Gy is strongly recommended for pain relief in painful bone metastases of breast cancer. However, the high rate of re-irradiation in the single-fraction group should be noted. Because patients with breast cancer often have a long-term prognosis, the indication for single-fraction RT should be carefully considered when risks such as spinal-cord compression are anticipated over the long-term.

**BQ12.** Is RT recommended for brain metastasis of breast cancer?

#### Statement

RT is the standard treatment.

**CQ8.** Is the addition of whole-brain irradiation recommended after stereotactic radiosurgery (SRS) for 1–4 brain metastases of breast cancer measuring < 3 cm?

#### Recommendation

It is weakly recommended not to add whole-brain irradiation after SRS [SoR: 3; SoE: moderate; consensus rate: 98% (40/41)].

Three RCTs comparing SRS with or without whole-brain irradiation were used to analyze OS and intracranial control [42–44]. For the cognitive function analysis, two RCTs that used the same evaluation methods were selected [43, 44].

##### OS

The OS did not differ in the two groups, 86% (178/208 patients) in the SRS alone group and 85% (165/195 patients) in the SRS + whole-brain irradiation group (HR 0.85, 95% CI 0.48–1.52, *p* = 0.59). A review of three RCTs of SRS ± whole-brain irradiation showed that SRS alone significantly improved OS in patients aged ≤ 50 years [45]. However, only a few patients with breast cancer were included in this study.

##### Intracranial control

The 1-year intracranial control rate was lower in the SRS alone group. The intracranial recurrence rate was 57% (113/200 patients) in the SRS alone group and 24% (44/184 patients) in the SRS + whole-brain irradiation group (RR 2.41, 95% CI 1.52–3.81, *p* = 0.0002), indicating that omitting whole-brain irradiation after SRS significantly increased the rate of intracranial recurrence. However, in two studies that reported neurological deaths [42, 44], there was no difference in the 1-year rate of neuropathic death between SRS with or without whole-brain irradiation.

##### Incidence of cognitive dysfunction

In a meta-analysis of the two studies using the Hopkins Verbal Learning Test-Revised (HVLT-R) [43, 44], cognitive dysfunction at 3 to 4 months was 53% (44/83) in the SRS alone group and 86% (51/59) in the SRS + whole-brain irradiation group (RR 0.53, 95% CI 0.25–1.15, *p* = 0.11), indicating that omitting whole-brain irradiation benefitted cognitive function. Furthermore, it should be noted that Chang et al. prematurely terminated the study before reaching the planned number of patients because of the higher rate of cognitive dysfunction in the group that received whole-brain irradiation. However, Aoyama et al. reported no difference in the median score of the Mini-Mental State Examination (MMSE) between the two groups at 1 year [42].

Although an appropriate method of assessing cognitive function has not been established, we weakly recommend not adding whole-brain irradiation after SRS because SRS alone may avoid cognitive dysfunction without compromising survival. The fact that SRS machines and MRI are widely available in Japan and that SRS can be performed as a standard treatment played a major role in this recommendation.

## Conclusions

This article described the radiation therapy section of the 2022 edition of the JBCS Clinical Practice Guidelines. These guidelines are expected to play a significant role in guiding decision-making by radiation oncologists, surgeons, physicians, other medical staff, and patients.

## Data Availability

Data sharing is not applicable to this article as no datasets were generated or analyzed during the current study.
